# Ergogenic Effect of Nitrate Supplementation in Clinical Populations: A Systematic Review and Meta-Analysis

**DOI:** 10.3390/nu16223832

**Published:** 2024-11-08

**Authors:** Cassandra C. Derella, Kara C. Anderson, Mary N. Woessner, Craig Paterson, Jason D. Allen

**Affiliations:** 1 Department of Kinesiology, University of Virginia, Charlottesville, VA 22903, USA; 2 Division of Endocrinology and Metabolism, School of Medicine, University of Virginia, Charlottesville, VA 22903, USA; 3 Institute for Health and Sport, Victoria University, Melbourne, VIC 3011, Australia; 4 Population Health Sciences, Bristol Medical School, University of Bristol, Bristol BS8 2PS, UK; 5 Division of Cardiovascular Medicine, School of Medicine, University of Virginia, Charlottesville, VA 22903, USA

**Keywords:** inorganic nitrate, clinical population, time to exhaustion, VO_2Peak_

## Abstract

**Background/Objectives:** Inorganic nitrate (NO_3_^−^) supplementation, via its conversion to nitric oxide (NO), has been purported to be ergogenic in healthy individuals. Many disease states are characterized by reduced NO bioavailability and are expected to derive a benefit from NO_3_^−^. This systematic review and meta-analysis evaluate the current literature on the ergogenic effect of NO_3_^−^ supplementation in individuals with cardiopulmonary and metabolic diseases (CPMD). **Methods:** Relevant databases were searched up to December 2023 for randomized, placebo-controlled crossover trials for aerobic exercise outcome variables with CPMD. **Results:** Twenty-two studies were included, and 46% reported ergogenic benefits of inorganic nitrate supplementation. NO_3_^−^ supplementation had no effect on aerobic performance with respect to maximal (SMD = 0.11, 95% CI: −0.12 to 0.34, *p* = 0.34) and submaximal (SMD = 0.16, 95% CI: −0.13 to 0.46, *p* = 0.27) TTE, VO_2peak_ (SMD = 0.002, 95% CI: −0.37 to 0.38, *p* = 0.99), or 6MW (SMD = 0.01, 95% CI: −0.29 to 0.28, *p* = 0.96). When the studies were limited to only cardiovascular disease conditions, NO_3_^−^ supplementation had trivial effects on aerobic performance with respect to Timed Trials (SMD = 0.14, 95% CI: −0.04 to 0.33, *p* = 0.13), VO_2_ (SMD = −0.02, 95% CI: −0.32 to 0.27, *p* = 0.87), and small effects on Distance Trials (SMD = 0.25, 95% CI: −0.18 to 0.69, *p* = 0.25). Sunset funnel plots revealed low statistical power in all trials. **Conclusions:** The results of this systematic review revealed that 46% of the individual studies showed a positive benefit from inorganic nitrate supplementation. However, the meta-analysis revealed a trivial effect on physical function in CPMD populations. This is likely due to the large heterogeneity and small sample sizes in the current literature.

## 1. Introduction

Inorganic nitrate (NO_3_^−^), a common component found in various vegetables and root crops, was generally considered biologically inert when consumed as part of the diet. However, recent research has shown that specific metabolic pathways in the human body can convert dietary NO_3_^−^ into nitric oxide (NO), a molecule with significant physiological roles, including vascular health, metabolic function, and immune response [1,2]. This conversion process involves the reduction of NO_3_^−^ to nitrite (NO_2_^−^) in the oral cavity and subsequently to NO by a variety of pathways involving single-electron transfer reactions with protons (H^+^) and hemeproteins (i.e., hemoglobin, myoglobin) during deoxygenation [3,4]. The discovery of this conversion pathway has opened new avenues for understanding the potential health benefits of dietary NO_3_^−^.

The interest in the ergogenic effects of NO_3_^−^ supplementation has expanded rapidly since 2007, when Larsen and colleagues showed that three days of dietary sodium nitrate (NaNO_3_^−^) resulted in a reduction in oxygen cost during submaximal cycling [5] and followed up to show that NO_3_^−^ supplementation resulted in lower maximal oxygen consumption (VO_2max_) and increased time to exhaustion in young males [6]. Studies have continued to suggest that NO_3_^−^, via its conversion to NO, may enhance exercise performance in healthy populations [7,8,9,10,11,12]. This enhancement is attributed to NO’s ability to improve mitochondrial efficiency, reduce oxygen cost during exercise, and increase tissue perfusion to active tissues [10,13]. Supporting this, a recent meta-analysis showed an ergogenic effect of NO_3_^−^ supplementation in recreationally active, young, healthy males [14], and an expert consensus statement (using the modified Delphi technique) [15] concluded that acute and chronic NO_3_^−^ supplementation is likely to produce ergogenic benefits during acute exercise in individuals with lower and more moderate aerobic fitness.

Most populations with cardiopulmonary and metabolic disease or risk factors, such as advanced age, hypertension, heart failure, and diabetes, are characterized by endothelial dysfunction and diminished bioavailable NO [16]. These individuals also have low levels of physical function and may be at risk of losing functional independence. Given NO’s critical role in maintaining vascular health and potentially ergogenic effects, it is logical to hypothesize that exogenous NO_3_^−^ supplementation might enhance NO bioavailability, leading to improvements in physical function (and overall health) in individuals with cardiopulmonary and/or metabolic disease, who may need it the most. 

The primary purpose of this systematic review and meta-analysis is to synthesize existing evidence and provide a detailed evaluation of the potential benefits and therapeutic implications of NO_3_^−^ supplementation for enhancing physical function in cardiopulmonary and metabolic disease populations. Given the multiple etiologies, pathologies, and phenotypes involved in this review and the prevalence of cardiovascular disease worldwide (CVD), we have a more circumscribed secondary purpose to explore whether NO_3_^−^ supplementation augments exercise tolerance in people with CVD.

## 2. Materials and Methods

This meta-analysis and systematic review are reported in accordance with PRISMA (Preferred Reporting Items for Systemic Reviews and Meta-Analyses) guidelines [17]. This review was not registered. 

**Literature Search:** Search terms and study criteria were all determined a prior to searches and were not altered throughout the search process. Electronic databases (Pubmed, Web of Science, and Scopus) were searched by two authors (CCD and KCA), with articles published through December 2023. The search used the following terms: (exercise AND nitrate). Reference lists of all relevant studies, along with reviews and book chapters, were also examined. Articles were limited to randomized controlled trials (RCT) in humans written in the English language. 

**Study Selection:** For this meta-analysis, the term ‘article’ is used synonymously with ‘study’ and ‘trial’ is the unit included in the meta-analysis. Articles often contained multiple eligible trials that comprised an intervention group and a comparable control. 

First, the titles and abstracts of articles were screened for eligibility. The following criteria were determined a priori for article inclusion: (1) any cardiopulmonary and metabolic diseases (not including obesity or hypertension as primary conditions), (2) adults over 18 years of age, (3) randomized, placebo-controlled crossover trial with single or double blinding, (4) inorganic and oral nitrate supplementation (sodium-nitrate, potassium-nitrate, and beetroot juice), (5) provided the dosage of nitrate given, (6) aerobic exercise outcome variable (time to exhaustion, VO_2peak_, graded exercise test, 6 min walk/maximal distance walked), (7) acute or chronic supplementation, and (8) data needed to be reported as mean with SD or SEM. Articles that solely investigated obesity were excluded as it is not a cardiometabolic disease (type 2 diabetes or metabolic disease with obesity were included). Hypertension alone was excluded as it is a condition and risk factor, not a cardiometabolic disease, and its relationship with nitrate supplementation has been extensively reviewed elsewhere. Full texts were reviewed of the remaining articles to determine eligibility. Two authors (CCD and KCA) independently completed the study selection, and disagreements were resolved by a third reviewer (JDA).

**Data Extraction and Bias Assessment:** For studies that met the inclusion criteria, the following data were extracted and tabulated: (1) author and publication year; (2) continuous variables: sample size, age, body mass index, relative VO_2Peak_, submaximal relative VO_2_, maximal time to exhaustion (TTE during an incremental maximal exercise test), submaximal TTE (fixed work load), distance walked during a 6 min walk test (6MW), time walked during an endurance shuttle walk test (ESWT), distance walked during an incremental shuttle walk test (ISWT), distance walked until claudication onset (COD), and time walked until claudication onset (COT), duration of the NO_3_^−^ treatment (if longer than one acute dose), NO_3_^−^ dosage in mmol and mmol/kg/day, timing of post-treatment testing (minutes after last dose), post-treatment plasma NO_3_^−^ and NO_2_^−^; and (3) categorical variables: sex, route of NO_3_^−^ administration, placebo utilized, mode of exercise test (cycle ergometer vs treadmill), and the clinical population. If data were not available in the articles, authors were contacted for data. In the case where the authors did not respond to follow up, means and standard deviations were extracted from relevant figures using ImageJ software (ImageJ 1.56g, Wayne Rasband and contributors, National Institute of Health, Bethesda, MD, USA) [18]. Extraction was completed independently by two authors (CCD and KCA), and the average was calculated.

Risk of bias was assessed using the Cochrane risk-of-bias tool for randomized trials (RoB 2), which includes the following domains: randomization, deviations from interventions, missing outcome data, measurement of outcome data, and results [19]. In each domain are signaling questions, where the risk of bias calculated from each domain is generated from an algorithm. Each study is scored as either “low risk”, “high risk”, or “some concern” of bias based on the answers to the signaling questions. Two authors (CCD and KCA) independently answered the signaling questions, and disagreements were resolved by a third reviewer (JDA).

**Narrative Synthesis:** Initially, a narrative synthesis of studies was conducted. Studies were listed in alphabetical order based on the disease condition. This summary table is provided as supplemental content (Table S1). 

**Meta-Analysis:** To support the narrative synthesis, a meta-analysis was performed using R Version 4.3.2, and the “metafor”, “metaviz”, and “ggplot2” packages 3.0.2, 0.3.1, and 3.3.5, respectively [20,21]. Significance levels for all hypothesis testing were set a prior at *p* = 0.05. Due to the different methods employed to assess each outcome measure, outcomes were converted to standard mean difference (SMD) to facilitate the meta-analysis. The SMDs of all analyses are expressed as Hedges g and are interpreted as follows: <0.20, 0.2, 0.5, and 0.8 are considered to represent trivial, small, moderate, and large effect sizes, respectively [22]. SMDs are reported with 95% confidence intervals (CI) unless otherwise noted.

Due to the typically small sample sizes of included studies, it was decided a prior that this meta-analysis would be performed on a minimum of 4 studies to avoid producing estimates based on minimal evidence. Thus, the SMD for each exercise outcome (TTE, submaximal TTE, VO_2Peak_, 6MW distance) with 4 or more trials was inputted into the model to determine the pooled effect for each exercise outcome. To investigate the impact of NO_3_^−^ supplementation in patients with CVD, a secondary analysis was performed only including CVD populations (heart failure, PAD, NICD) and exercise tolerance metrics with similar outcomes/units were collapsed into groups: (1) timed trials (TTE, submaximal TTE, COT), (2) oxygen consumption/VO_2_ (peak, submaximal, relative and absolute values), and (3) distance trials (6MW, ISWT, COD, ESWT). A random effects model with restricted maximum likelihood estimation was used. 

Cook’s distance was used to identify potentially influential trials. If a trial was identified as potentially influential, the robustness of the pooled result was assessed by removing the influential trial(s) and re-examining the observed effect and heterogeneity. Additionally, sunset plots were used to assess small study effects and the observed power of included trials within each analysis. 

Moderator analysis was performed to determine whether sex impacted the overall pooled effects. Meta-regressions were also used to determine if the following continuous variables impacted the pooled correlation coefficient: age, NO_3_^−^ dose, treatment duration, and timing of NO_3_^−^ supplementation before post-testing.

## 3. Results

### 3.1. Literature Search

**Figure 1:**
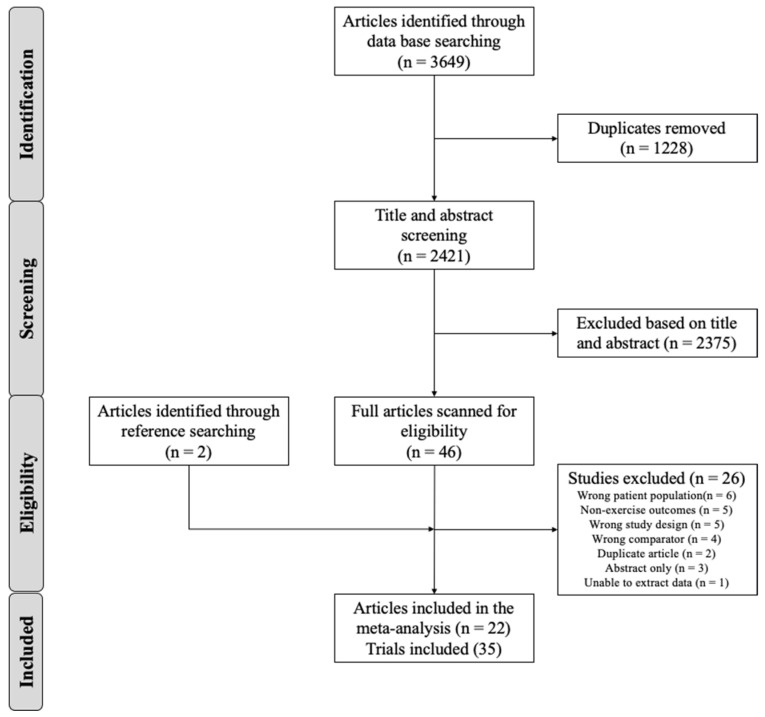
PRISMA Diagram.

The PRISMA flow diagram outlining this process is presented in Figure 1. The initial search identified 3649 articles found via the database searches. After title and abstract screening, 2375 articles were excluded, leaving 46 eligible articles, with an additional 2 articles found through reference list searching. Following full-text review, 22 articles met the inclusion criteria, with 35 acceptable trials [23,24,25,26,27,28,29,30,31,32,33,34,35,36,37,38,39,40,41,42,43,44]. 

### 3.2. Study Characteristics: Narrative Review

Studies were published between 2011 and 2023. A total of 394 participants were included in the trials, with 60% of the population being composed of males. One study only included males [29] and one did not report sex [41]; however, most included both males and females (k = 20) [23,24,25,26,27,29,30,31,32,33,34,35,36,37,38,39,40,41,42,43,44]. The primary clinical population of studies included chronic obstructive pulmonary disease (COPD, k = 8), heart failure with reduced ejection fraction (HFrEF, k = 4), peripheral artery disease (PAD, k = 4), heart failure with preserved ejection fraction (HFpEF, k = 2), non-ischemic dilated cardiomyopathy (NIDC, k = 1), chronic kidney disease (CKD, k = 1), angina (k = 1), and type 2 diabetes (T2D, k = 1). The primary exercise performance metric of trials included time to exhaustion during an incremental exercise test (TTE; k = 7), submaximal TTE during a fixed workload (k = 5), relative VO_2Peak_ (k = 5), relative submaximal VO_2_ (k = 3), absolute submaximal VO_2_ (k = 1; weight not reported), 6 min walk distance (6MW; k = 5), claudication onset time (COT; k = 3), claudication onset distance (COD; k = 1), incremental shuttle walk test (ISWT; k = 3), and the endurance shuttle walking test (ESWT; k = 2). NO_3_^−^ supplementation was primarily ingested via beetroot juice (k = 21) and NaNO_3_^−^ (k = 1) and ingested 45–180 min prior to exercise with a concentration between 4.8 mmol and 16 mmol for 1–14 days. Two studies provided dosing according to body mass (0.11 mmol/kg), and the average dosing equated to ~8.47mmol NO_3_^−^ (calculated by 0.11mmol/kg × 7.0 kg [average body mass in the trials]) [36,37]. Of the 35 exercise trials, 16 (46%) observed statistically significant improvements in exercise parameters, while 19 (54%) observed no changes in exercise outcomes, and no studies reported worsened performance with NO_3_^−^ supplementation compared to placebo. 

Due to the limited number of trials in each reported exercise performance metric, only 22 (of 35) trials were analyzed in the first part of the meta-analysis portion of this report. The exercise parameters and populations included TTE (k = 7: HFrEF = 2, HFpEF = 1, PAD = 2, CKD = 1, Angina = 1), submaximal TTE (k = 5: HFpEF = 2, HFrEF = 1, COPD = 2), VO_2Peak_ (k = 5: HFrEF = 2, HFpEF = 1, PAD = 1, CKD = 1), and 6MW distance (k = 5: T2D = 1, COPD = 2, PAD = 1, HFrEF = 1). In consideration that 50% of the studies included a form of CVD, and because these patients develop multiple peripheral tissue abnormalities that may greatly benefit from NO_3_^−^ supplementation [45], a secondary analysis was performed to assess NO_3_^−^ supplementation effects on exercise tolerance in CVD. This summary table is provided as supplemental content (Table S2).

### 3.3. Risk of Bias

Overall, studies were deemed to have a low risk of bias (Figure S1). All studies utilized blinded and placebo-controlled approaches. Of the 22 studies included, only one had some concerns due to performing three rounds of walking prior to the primary outcome of 6MW distance [40]. Although not indicated by the RoB2 analysis, one study reported an increase in plasma NO_2_^−^ following their placebo treatment similar to that found following their NO_3_^−^-rich dietary intervention (NO_3_^−^-rich vegetables) [42]. This raised some concerns about the validity of the results, as no differences were observed in plasma NO_2_^−^ across groups, and therefore, the lack of differences in the primary outcomes of TTE and COT cannot be attributed to treatment differences. With this in mind, we performed the first part of the analysis, excluding this trial. However, its exclusion did not change the overall results (Table S3), and therefore, all further results include all trials unless noted.

### 3.4. Pooled Analysis

#### 3.4.1. TTE

**Figure 2:**
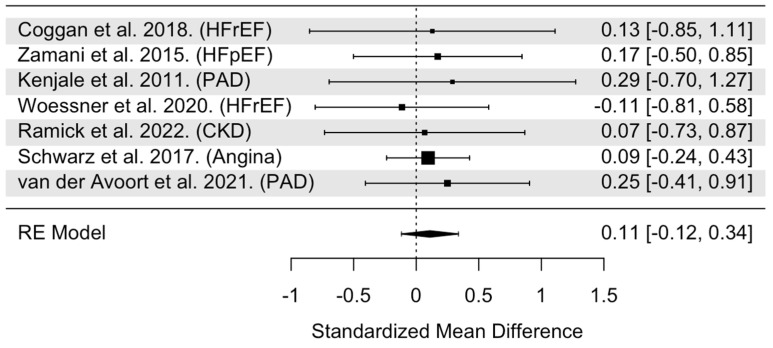
Forest plot of overall model for TTE (during an incremental workload exercise test) trial [24,30,38,39,42,43,44]. Abbreviations: HFrEF, heart failure with reduced ejection fraction; HFpEF, heart failure with preserved ejection fraction; PAD, peripheral arterial disease; CKD, chronic kidney disease.

The overall model indicated that NO_3_^−^ supplementation had statistically non-significant effects on maximal TTE (SMD = 0.11, 95% confidence interval [CI]: −0.12 to 0.34, *p* = 0.34, Figure 2). Heterogeneity was found to be statistically non-significant (Cochran’s Q = 0.75, df = 6, I^2^ = 0.00%, *p* = 0.99). Cook’s distance identified two individual trials as potentially influential (Figure S2) [39,43]. Within these two trials, one had a large sample size (*n* = 70) compared to other trials (median *n* = 16) [39]. However, it was unclear as to why the other trial was visually marked beyond the trial, having a negative SMD (−0.11) and not reaching a 400 nM plasma NO_2_^−^ threshold compared with the other trials [43]. When these trials were removed from the pooled analysis, the effects of NO_3_^−^ supplementation remained statistically insignificant on maximal TTE (SMD = 0.18, 95% CI: −0.17 to 0.53 *p* = 0.30). Visual inspection of the funnel plot revealed no asymmetry and a median power of 6.1% of the included trials (Figure S3). Moderator data are presented in Table 1. The analyses revealed statistically non-significant moderation by age (*p* = 0.71), sex (*p* = 0.51), NO_3_^−^ dose (*p* = 0.79), treatment duration (*p* = 0.64), and timing of NO_3_^−^ supplementation (*p* = 0.71).

#### 3.4.2. Submaximal TTE

**Figure 3:**
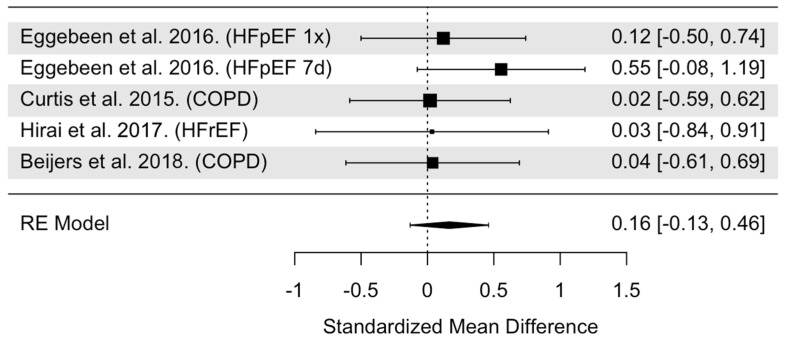
Forest plot of overall model for submaximal TTE (fixed workload exercise test) trials [23,26,27,29]. Abbreviations: HFpEF, heart failure with preserved ejection fraction; COPD, chronic obstructive pulmonary disease; HFrEF, heart failure with reduced ejection fraction; 1x = acute treatment; 7d = 7-day treatment.

The overall model indicated that NO_3_^−^ supplementation had statistically non-significant effects on submaximal TTE (SMD = 0.16, 95% CI: −0.13 to 0.46, *p* = 0.27, Figure 3). Heterogeneity was found to be statistically non-significant (Cochrane’s Q = 1.94, df = 4, I^2^ = 0.00%, *p* = 0.75). Cook’s distance identified one influential trial (Figure S4) [27]. It was unclear as to why this trial had such an effect on the model beyond it being the only trial to report a significant increase in submaximal TTE and had the greatest SMD (0.55) compared to the other trials. When this trial was removed from the pooled analysis, the effects of NO_3_^−^ supplementation remained statistically insignificant on submaximal TTE (SMD = 0.06, 95% CI: −0.28 to 0.29, *p* = 0.74). Visual inspection of the funnel plot revealed no asymmetry and a median power of 8.0% of the included trials (Figure S5). Moderator data is presented in Table 1. The subgroup analysis revealed statistically non-significant moderation by age (*p* = 0.51), sex (*p* = 0.33), NO_3_^−^ dose (*p* = 0.49), treatment duration (*p* = 0.58), and timing of NO_3_^−^ supplementation (*p* = 0.62).

#### 3.4.3. VO_2Peak_

**Figure 4:**
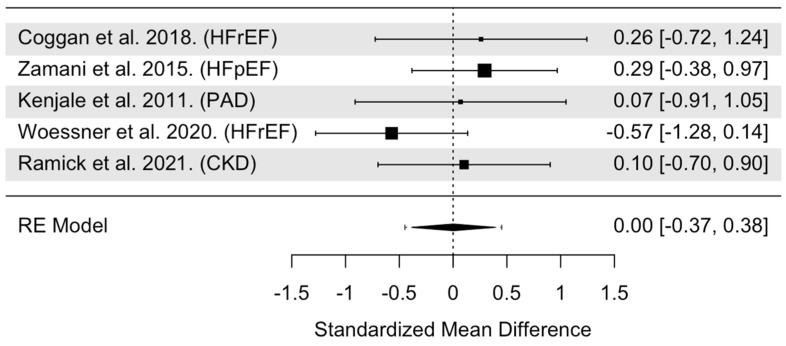
Forest plot of overall model for VO_2_Peak trials [24,30,38,43,44]. Abbreviations: HFrEF, heart failure with reduced ejection fraction; HFpEF, heart failure with preserved ejection fraction; PAD, peripheral arterial disease; CKD, chronic kidney disease.

The overall model indicated that NO_3_^−^ supplementation had a statistically insignificant effect on relative VO_2Peak_ (SMD = 0.002, 95% CI: −0.37 to 0.38, *p* = 0.99, Figure 4). Heterogeneity was found to be statistically non-significant (Cochrane’s Q = 3.58, df = 4, I^2^ = 8.59%, *p* = 0.47). Cook’s distance identified one trail as an influential trial (Figure S6) [43]. Compared with the other VO_2Peak_ trials, this trial was the only one in this group that utilized a subacute (7-day) treatment model (compared to single, acute doses in the other four trials). When this trial was removed from the pooled analysis, the effects of NO_3_^−^ supplementation remained statistically insignificant on relative VO_2Peak_ (SMD = 0.20, 95% CI: −0.22 to 0.61, *p* = 0.35). Visual inspection of the funnel plot revealed no asymmetry in median power of 5.0% of the included trials (Figure S7). Moderator data are presented in Table 1. The subgroup analysis revealed statistically insignificant moderation by age (*p* = 0.85), sex (*p* = 0.52), NO_3_^−^ dose (*p* = 0.22), and timing of NO_3_^−^ supplementation (*p* = 0.31). Four out of five of the trials in this pooled analysis were single acute treatments; therefore, treatment duration was not evaluated.

#### 3.4.4. 6MW Distance 

**Figure 5:**
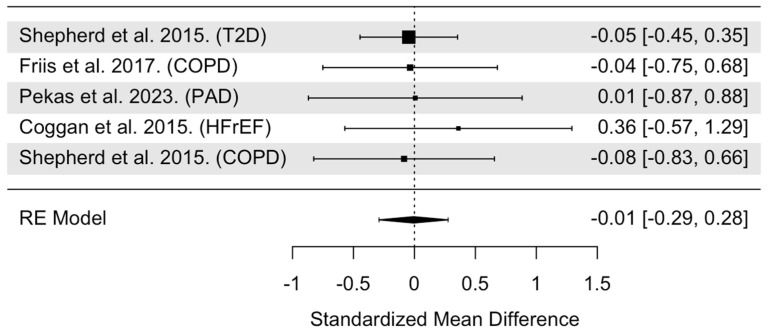
Forest plot of overall model for 6MW trials. Abbreviations: T2D, type 2 diabetes; COPD, chronic obstructive pulmonary disease; HFrEF, heart failure with reduced ejection fraction.

The overall model indicated that NO_3_^−^ supplementation had statistically non-significant effects on the distance walked during a 6MW test (SMD = −0.01, 95% CI: −0.29 to 0.28, *p* = 0.96, Figure 5). Heterogeneity was found to be statistically non-significant (Cochrane’s Q = 0.68, df = 4, I^2^ = 0.00%, *p* = 0.95). Cook’s distance identified two trials as potentially influential (Figure S8) [25,40]. Within these two trials, one had a large effect, likely due to a large sample size (*n* = 48) [40], while the other trial [25] had the largest SMD (0.36) compared to the other 6MW distance trials [28,36,41]. When these two trials were removed from the pooled analysis (leaving only three trials for comparison), the effects of NO_3_^−^ supplementation remained statistically insignificant on the 6MW distance (SMD = −0.04, 95% CI: −0.49 to 0.40, *p* = 0.85). Visual inspection of the funnel plot revealed no asymmetry and a median power of 5.0% of the included trials (Figure S9). Moderator data are presented in Table 1. The analysis revealed statistically non-significant moderation by age (*p* = 0.59), sex (*p* = 0.76), NO_3_^−^ dose (*p* = 0.72), treatment duration (*p* = 0.75), and timing of NO_3_^−^ supplementation (*p* = 0.61).

### 3.5. Pooled Analysis in CVD

#### 3.5.1. Timed Exercise Trials in CVD 

**Figure 6:**
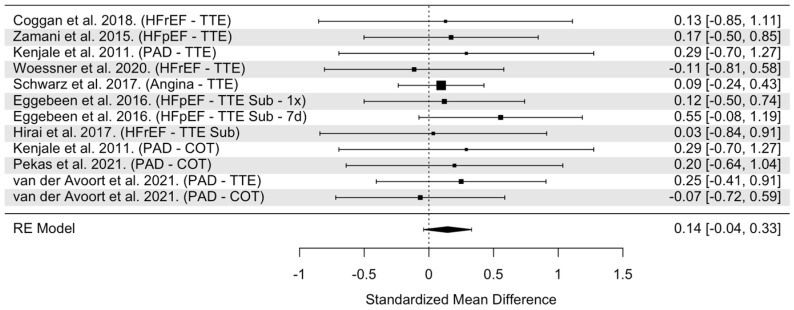
Forest plot of overall model for Timed Exercise Trials in CVD populations only [24,27,29,30,37,39,42,43,44]. Abbreviations: HFrEF, heart failure with reduced ejection fraction; HFpEF, heart failure with preserved ejection fraction; PAD, peripheral arterial disease; TTE, time to exhaustion during an incremental workload exercise/max test; TTE Sub, time to exhaustion during a fixed workload exercise test; COT, claudication onset time; 1x = acute treatment; 7d = 7-day treatment.

The overall model indicated that NO_3_^−^ supplementation had statistically non-significant effects on timed exercise trials in CVD (SMD = 0.14, 95% confidence interval [CI]: −0.04 to 0.33, *p* = 0.13, Figure 6). Heterogeneity was found to be statistically non-significant (Cochrane’s Q = 3.00, df = 11, I^2^ =0 0.00%, *p* = 0.99). Cook’s distance indicated three trials as potentially influential (Figure S10) [27,42,43]. Possible rationale as to why these trials were flagged as influential include negative SMD [42,43] and a large sample size [39]. When these trials were removed from the pooled analysis, the effects of NO_3_^−^ supplementation remained statistically insignificant on timed exercise trials (SMD = 0.17, 95% CI: −0.14 to 0.47, *p* = 0.29). Visual inspection of the funnel plot revealed no asymmetry and a median power of 7% of included trials (Figure S11).

#### 3.5.2. VO_2_ in CVD

**Figure 7:**
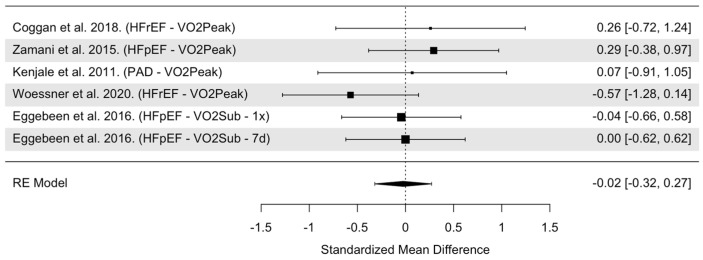
Forest plot of overall model for VO_2_ in CVD populations only [24,27,30,43,44]. Abbreviations: HFrEF, heart failure with reduced ejection fraction; HFpEF, heart failure with preserved ejection fraction; PAD, peripheral arterial disease; VO_2_Peak, maximal/peak oxygen consumed during an incremental workload exercise test; VO_2_Sub, maximal oxygen consumed during a submaximal/fixed workload exercise test; 1x = acute treatment; 7d = 7-day treatment.

The overall model indicated that NO_3_^−^ supplementation had statistically non-significant effects on VO_2_ in people with CVD (SMD = −0.02, 95% confidence interval [CI]: −0.32 to 0.27, *p* = 0.87, Figure 7). Heterogeneity was found to be statistically non-significant (Cochrane’s Q = 3.51, df = 5, I^2^ = 0.00%, *p* = 0.62). Cook’s distance identified one trial as a potentially influential study (Figure S12), which was the only trial with a large negative SMD [43]. When this trial was removed from the pooled analysis, the effects of NO_3_^−^ supplementation remained statistically insignificant in people with CVD (SMD = 0.09, 95% CI: −0.23 to 0.42, *p* = 0.58). Visual inspection of the funnel plot revealed no asymmetry and a median power of 5.1% of included trials (Figure S13). 

#### 3.5.3. Distance Exercise Trials in CVD

**Figure 8:**
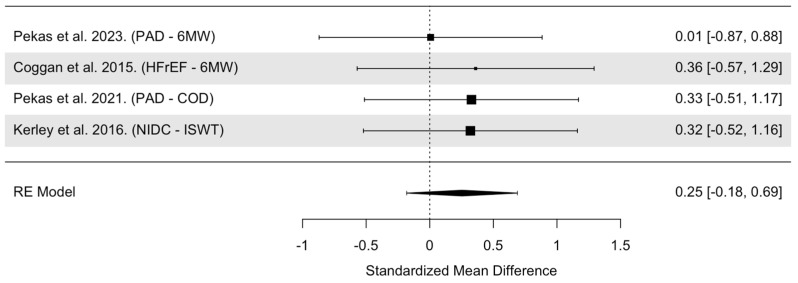
Forest plot of overall model for Distance Exercise Trials in CVD populations only [25,33,36,37]. Abbreviations: PAD, peripheral arterial disease; HFrEF, heart failure with reduced ejection fraction; NIDC, non-ischemic dilated cardiomyopathy; 6MW, 6 min walk test/distance; COD, claudication onset distance (distance walked to the onset of claudication pain); ISWT, incremental shuttle walk test.

The overall model indicated that NO_3_^−^ supplementation had statistically non-significant small effects on distance exercise trials in people with CVD (SMD = 0.25, 95% confidence interval [CI]: −0.18 to 0.69, *p* = 0.25, Figure 8). Heterogeneity was found to be statistically non-significant (Cochrane’s Q = 0.41, df = 3, I^2^ = 0.00%, *p* = 0.94). There were only four distance trials that included people with CVD. Visual inspection of the funnel plot revealed no asymmetry and a median power of 8.9% of the included trials (Figure S14). 

## 4. Discussion

Since the initial study showing an ergogenic benefit of acute inorganic NO_3_^−^ supplementation for pain-free and peak treadmill ambulation in patients with PAD in 2011 [30], there has been a growing interest in therapeutic applications of inorganic NO_3_^−^ to increase physical function in clinical populations. Over two decades later, the opinions of twelve experts in the use of dietary NO_3_^−^ as an ergogenic aid concluded that individuals with low aerobic fitness who consumed 8–16 mmol inorganic NO_3_^−^ were most likely to achieve an ergogenic benefit [15]. However, there was insufficient evidence to form a consensus with regard to the effects of inorganic NO_3_^−^ supplementation on performance in clinical populations [15].

The results of this systematic review and meta-analysis of 35 trials (from 22 studies) suggest a statistically non-significant effect of inorganic NO_3_^−^ supplementation on TTE, VO_2Peak_, or 6MW distance in participants with cardiopulmonary and metabolic populations. The results were the same when limited to studies that included participants with CVD only. An inspection of the individual study findings shows that 16 trials (46%) report a statistically significant increase in the primary physical function outcome compared with the remaining 19 trials (54%), which showed no statistically significant difference. These values were 12 (55%) favorable and 10 neutral when examining trials that included only populations with CVD. The overall low number of trials and the small sample sizes (median sample size = 15, range 8 to 70, with only three trials including more than 20 participants [26,39,40]) are likely major contributors to the trivial effect size and lack of statistical significance in this meta-analysis. In fact, many of the studies in this analysis are likely underpowered to detect ergogenic effects of NO_3_^−^ supplementation if they were present. The inclusion of multiple trials with fewer than 20 participants weakens the overall strength of the current analysis.

Our statistically non-significant findings have similar effect sizes to those of a recent systematic review and meta-analysis of studies in young, healthy subjects, which showed a statistically significant effect of inorganic NO_3_^−^ supplementation on exercise performance [14]. This was despite less than one-third (35/80, 32%) of the individual studies showing a significant benefit in performance [14]. The major discrepancies between the two analyses are the populations studied (healthy vs. clinical) and the number of studies included in each analysis. The literature in young, healthy subjects included 80 studies (initial *n* = 2033 as of August 2019), whereas the number of reports in clinical populations is sparse, and our analysis was limited to 22 articles/studies (initial *n* = 3649 through December 2023). Furthermore, as suggested by the funnel plots (Supplementary Data), the trials included in this analysis were also largely not powered to determine the effect of NO_3_^−^ supplementation on the physical function outcome. While it is physiologically logical that populations with reduced endogenous NO bioavailability, such as those with cardiometabolic diseases, may benefit the most from exogenous supplementation when compared with healthy young subjects [15], to date, the data included in this current analysis do not support that hypothesis. More large studies in this area are necessary to definitively answer the question at hand. 

From an analytical perspective, the scarcity of studies on NO_3_^−^ supplementation in clinical populations necessitated a condensing of studies, including participants with different etiology, pathophysiology, and phenotype. This may have masked any disease-specific conditions that may be more likely to benefit from supplementation. For example, four out of the five trials evaluating pain-free ambulation in PAD showed significant benefits. The fifth study (two trials) showed mixed results but had no differences in plasma NO_2_^−^ values between the treatment and placebo groups, suggesting that inorganic NO_3_^−^ supplementation was not different/effective [42]. In contrast, in ten trials in patients with COPD, only three found a significant benefit, seven were equivocal, and only one trial investigated T2D, leaving metabolic disease understudied. While it would be of great benefit to perform a more focused meta-analysis on one or two specific diseases, the scarcity of the current literature on each disease prevents this. Larger scale studies investigating PAD and other individual cardiovascular and metabolic diseases may provide greater insight into the ergogenic benefit of NO_3_^−^ supplementation in clinical populations. 

The dose and mode of NO_3_^−^ supplementation employed across trials were also varied. Although most trials utilized beetroot juice (k = 32, 91%) with doses greater than 8 mmol (k = 20, 57%), dosages ranged from 1 acute dosage of 6.1 to 12.9 mmol given for 2–14 days. Different doses for different lengths of time can vary outcomes, making the comparison across studies difficult. With this in consideration, we included the dose and length of supplementation as a moderator; however, no effect was observed. Given that the oral microbiome has been shown to be vital in the conversion of NO_3_^−^ to NO_2_^−^, and subsequently NO, it may be that patients with chronic disease may have a disrupted oral microbiome, which could require higher or longer doses of NO_3_^−^ compared with what is established for younger and healthy populations. To the best of our knowledge, this hypothesis is untested but possible, as it is observed with other pharmaceutical approaches [46] and in the exercise intensity literature [4,47]. We have previously published a mini-review on this topic specific to heart failure [48] in which we suggest a potential disruption in the nitrate–nitrite reduction pathway in HFrEF compared with HFpEF populations and healthy controls. Such derangements in specific clinical cohorts could contribute to different effects of inorganic nitrate supplementation on physical function.

## 5. Conclusions

Despite the expert consensus that individuals with low aerobic fitness may benefit from NO_3_^−^ supplementation, the results of this systematic review and meta-analysis are inconclusive for the effect of inorganic NO_3_^−^ supplementation on TTE, VO_2Peak_, or 6MW distance in participants with cardiopulmonary and metabolic populations. Within the literature on an individual basis, there is a clear split in the data, where half of the studies report a benefit and half show no effect (but no determent). This is likely due to the overall dearth of studies in clinical populations, diverse disease conditions (which may not all respond equally), small subject numbers, varying dosing regimens, and different outcome measures within these studies. Inorganic NO_3_^−^ supplementation is a relatively inexpensive, easily administered approach to potentially increase physical function in cardiometabolic disease populations, and further large-scale trials are warranted to advance the feasibility of NO_3_^−^ supplementation for these populations.

## Figures and Tables

**Table 1:** Moderator analysis data.

Moderator Variable	*p*-Value	Comparison
**Maximal Time to Exhaustion (** **s** **)**		
Age	0.71	Meta-regression (β = 0.010, 95% CI = −0.040 to 0.059)
Sex (%Male)	0.51	Males (ES = −0.007, 95% CI = −0.029 to 0.014)
NO_3_^−^ Dose	0.79	Meta-regression (β = 0.031, 95% CI = −0.202 to 0.264)
Treatment Duration (Days)	0.64	Meta-regression (β = −0.015, 95% CI = −0.079 to 0.048)
Timing (min before ex)	0.71	Meta-regression (β = 0.002, 95% CI = −0.009 to 0.014)
**Submaximal Time to Exhaustion (s)**		
Age	0.51	Meta-regression (β = 0.053, 95% CI = −0.105 to 0.210)
Sex (%Male)	0.33	Males (ES = −0.004, 95% CI = −0.013 to 0.005)
NO_3_^−^ Dose	0.49	Meta-regression (β = −0.038, 95% CI = −0.145 to 0.069)
Treatment Duration (Days)	0.58	Meta-regression (β = 0.025, 95% CI = −0.063 to 0.113)
Timing (min before ex)	0.62	Meta-regression (β = −0.001, 95% CI = −0.006 to 0.004)
**Maximal VO_2_ (ml/kg/min)**		
Age	0.85	Meta-regression (β = −0.008, 95% CI = −0.095 to 0.079)
Sex (%Male)	0.52	Males (ES = −0.009, 95% CI = −0.037 to 0.019)
NO_3_^−^ Dose	0.22	Meta-regression (β = −0.094, 95% CI = −0.241 to 0.052)
Timing (min before ex)	0.31	Meta-regression (β = 0.0004, 95% CI = −0.014 to 0.014)
**6-Minute Walk (m)**		
Age	0.59	Meta-regression (β = −0.027, 95% CI = −0.126 to 0.072)
Sex (%Male)	0.76	Males (ES = −0.004, 95% CI = −0.033 to 0.024)
NO_3_^−^ Dose	0.72	Meta-regression (β = 0.020, 95% CI = −0.087 to 0.127)
Treatment Duration (Days)	0.75	Meta-regression (β = −0.025, 95% CI = −0.178 to 0.127)
Timing (min before ex)	0.61	Meta-regression (β = −0.002, 95% CI = −0.009 to 0.005)

## Data Availability

Some or all datasets generated during and/or analyzed during the current study are not publicly available but are available from the corresponding author on reasonable request.

## Supplementary Materials

The following supporting information can be downloaded at: https://www.mdpi.com/article/10.3390/nu16223832/s1. Supplemental data are uploaded in a secondary form for review. Table S1. Study characteristics. Table S2. Study characteristics for studies in CVD only. Table S3. Pooled analysis excluding de Avoort et al. 2021. Figure S1. Risk of Bias (ROB) 2 Bias Assessment. Figure S2. TTE Cook’s Distance depicting Trials #5 (Woessner et al., 2020) [43] and Trial #6 (Schwartz et al., 2017) [39] as either outlying or influential trials. Figure S3. TTE Funnel Plot. The observed outcome is SMD which is plotted against the standard error. Figure S4. Submaximal TTE Cook’s Distance depicting Trial #4 (Eggenbeen et al., 2016 7d treatment trial) [27] as an outlying or influential trial. Figure S5. Submaximal TTE Funnel Plot. The observed outcome is SMD which is plotted against the standard error. Figure S6. VO_2_Peak Cook’s Distance depicting Trial #4 (Woessner et al., 2020) [43] as an outlying or influential trial. Figure S7. VO_2_Peak Funnel Plot. The observed outcome is SMD which is plotted against the standard error. Figure S8. 6MW Distance Cook’s Distance depicting Trials #1 (Coggan et al., 2015) [25] and #4 (Shepherd et al., 2015) [40] as outlying or influential studies. Figure S9. 6MW Distance Funnel Plot. The observed outcome is SMD which is plotted against the standard error. Figure S10. Timed Exercise Trials in CVD Cook’s Distance depicting Trial #4 (Woessner et al., 2020) [43], #5 (Schwarz et al., 2017) [39], #7 (Eggebeen et al. 2016) [27] and #12 (van der Avoort et al., 2021) [42] as outlying or influential trials. Figure S11. Timed Exercise Trials Funnel Plot. The observed outcome is SMD which is plotted against the standard error. Figure S12. VO_2_ in CVD Cook’s Distance depicting Trial #4 (Woessner et al., 2020) [43] as an influential trial. Figure S13. VO_2_ Trials Funnel Plot. The observed outcome is SMD which is plotted against the standard error. Figure S14. Distance Exercise Trials Funnel Plot. The observed outcome is SMD which is plotted against the standard error.
