# Supplementary material for: Ergogenic Effect of Nitrate Supplementation in Clinical Populations: A Systematic Review and Meta-Analysis
Source: Nutrients. 2024 Nov 8;16(22):3832. doi: 10.3390/nu16223832 (PMC11597481; doi:10.3390/nu16223832)
Supplement: Supplementary file 1 [file nutrients-16-03832-s001.zip › nutrients-3286163-supplementary.pdf]

Table S1. Study characteristics.

	Participants				Exercise		Nitrate Supplementation					
**Trial**	**Pop.**	**n (%Males)**	**Age (years)**	**BMI (kg/m^2)**	**Mode**	**Variable(s)**	**Mode**	**Dose (mmol)**	**Days Taken**	**Last Dose (min)**	**Post NO_3^- (µM)**	**Post NO_2^- (nM)**
Schwarz et al. 2017	Angina	70 (74%)	67±8	28.6±4.0	Tread	TTE (sec)	$NaNO_3^-$	7	8.5	150	297.6±164.3	552±320
Ramick et al. 2021	CKD	12 (75%)	60±16	32.0±5.0	Cycle	VO_2Peak (ml/min/kg); TTE (sec)	BRJ	12.6	1	150	-	-
Beijers et al. 2018	COPD	18 (72.2%)	67±8	25.93.4	Cycle	TTE Submax (sec); VO_2 Submax (ml/min/kg)	$NaNO_3^-$	8	7	150	133±106	245±165
Curtis et al. 2015	COPD	21 (76%)	68±7	25.2±5.5	Cycle	TTE Submax (sec)	BRJ	12.9	1	180	820.2±187.7	1570±980
Friis et al. 2017	COPD	15 (60%)	63±13	25.5±4.8	Walking	6MW (m)	BRJ	8.6	7	150	-	538.5±722.6
Kerley et al. 2015	COPD	11 (45%)	69±7	27.3±6.4	Walking	ISWT (m/sec)	BRJ	12.9	1	180	508±167	751±632
Kerley et al. 2018	COPD	8 (62%)	63±7	25.6±5.1	Walking	ISWT (m/sec)	BRJ	12.9	14	150	1046±1201	1713.2±576.6
Leong et al. 2015	COPD	19 (26%)	67±8	29.1±6.5	Walking	ESWT (m)	BRJ	4.8	3	60	-	-
Pavitt et al. 2022	COPD	20 (60%)	68±9	25.2±4.7	Walking	ESWT (m)	BRJ	12.9	1	180	-	-
Shepherd et al. 2015	COPD	14 (n/a)	65±8	29.0±8.0	Walking	6MW (m); VO_2 Submax (ml/min)	BRJ	6.77	2	180	215±84	-
Eggenbeen et al. 2016	HFpEF	20 (15%)	69±7	32.9±5.6	Cycle	TTE Submax (sec); VO_2 Submax (ml/min/kg)	BRJ	6.1	1 or 7	45	362±158; 461±229	810±910; 780±520
Zamani et al. 2015	HFpEF	17 (88%)	66±9	36.4±5.4	Cycle	VO_2Peak (ml/min/kg); TTE (sec)	BRJ	12.9	1	180	-	-
Coggan et al. 2015	HFrEF	9 (55%)	57±10	29.1±6.6	Walking	6MW (m)	BRJ	11.2	1	90	492.3±48.5	360±18

Coggan et al. 2018	HFrEF	8 (75%)	52±14	33.1±9.9	Cycle	TTE (sec); VO_2Peak (ml/min/kg)	BRJ	11.2	1	90	266.6±113.7	111±41
Hirai et al. 2017	HFrEF	10 (100%)	63±16	31±7	Cycle	TTE Submax (sec)	BRJ	6.45	9	90	-	-
Woessner et al. 2020	HFrEF	16 (94%)	63±14	31.8±8.4	Tread	VO_2Peak (ml/min/kg); TTE (sec)	BRJ	16	7	150	1084.3±471.8	342.1±234.3
Kerley et al. 2016	NIDC	11 (63%)	56±11	32.5±4.6	Walking	ISWT (m/sec)	BRJ	12.9	2	180	-	-
Kenjale et al. 2011	PAD	8 (50%)	67±13	28.6±5.8	Tread	TTE (sec); VO_2Peak (ml/min/kg); COT (sec)	BRJ	8.06	1	180	443.9±85.0	942.9±826.6
Pekas et al. 2021	PAD	11 (45%)	70±7	29.1±6.4	Tread	COD (m); COT (sec)	BRJ	0.11 (mmol/kg)	1	60	-	-
Pekas et al. 2023	PAD	10 (40%)	70±9	29.3±6.6	Tread	6MW (m)	BRJ	0.11 (mmol/kg)	1	60	-	-
van der Avoort et al. 2021	PAD	18 (61%)	73±8	25.0±3.0	Tread	TTE (sec); COT (sec)	BRJ	6.5	1	180	494±110	1353±1316
Shepherd et al. 2015	T2D	48 (73)	63±7	30.2±2.9	Walking	6MW (m)	BRJ	6.43	3.5	180	319±90	1065±607

Abbreviations: Pops. = the populations investigated in each study: COPD, chronic obstructive pulmonary disease; HFrEF, heart failure with reduced ejection fraction, HFpEF, heart failure with preserved ejection fraction; PAD, peripheral arterial disease; NIDC, non-ischemic dilated cardiomyopathy; and CKD, chronic kidney disease. Modes = forms of exercise modality used to evaluate fitness: cycle, cycle ergometer; tread, treadmill walking ramp or fixed workload; and walking, a walking field test. Variables = exercise outcomes assessed: TTE Submax, time to exhaustion during a fixed workload; VO_2 Submax, oxygen consumed during a fixed workload test; VO_2Peak, maximal oxygen consumed during an incremental test; TTE, time to exhaustion during an incremental maximal exercise test; 6MW, distance walked during a 6-minute walk, ESWT, time walked during an endurance shuttle walk test, ISWT, distance walked during an incremental shuttle walk test; COD or COT, distance or time walked until claudication pain. Mode = NO_3^- supplementation used: $NaNO_3^-$, sodium nitrate, or BRJ, beet root juice. Days taken = the length of treatment in days. Last Dose = the time in minutes the last dose was consumed before post-testing.

Supplementary Table 2.

Table S2. Study characteristics for studies in CVD only.

	Participants				Exercise		Nitrate Supplementation					
**Trial – CVD Only**	**Pop.**	**n (%Males)**	**Age (years)**	**BMI (kg/m^2)**	**Mode**	**Variable(s)**	**Mode**	**Dose (mmol)**	**Days Taken**	**Last Dose (min)**	**Post NO_3^- (µM)**	**Post NO_2^- (nM)**
Coggan et al. 2015	HFrEF	9 (55%)	57±10	29.1±6.6	Walking	6MW (m)	BRJ	11.2	1	90	492.3±48.5	360±18
Coggan et al. 2018	HFrEF	8 (75%)	52±14	33.1±9.9	Cycle	TTE (sec); VO_2Peak (ml/min/kg)	BRJ	11.2	1	90	266.6±113.7	111±41
Eggenbeen et al. 2016	HFpEF	20 (15%)	69±7	32.9±5.6	Cycle	TTE Submax (sec); VO_2 Submax (ml/min/kg)	BRJ	6.1	1 or 7	45	362±158; 461±229	810±910; 780±520
Hirai et al. 2017	HFrEF	10 (100%)	63±16	31±7	Cycle	TTE Submax (sec)	BRJ	6.45	9	90	-	-
Kenjale et al. 2011	PAD	8 (50%)	67±13	28.6±5.8	Tread	TTE (sec); VO_2Peak (ml/min/kg); COT (sec)	BRJ	8.06	1	180	443.9±85.0	942.9±826.6
Kerley et al. 2016	NIDC	11 (63%)	56±11	32.5±4.6	Walking	ISWT (m/sec)	BRJ	12.9	2	180	-	-
Pekas et al. 2021	PAD	11 (45%)	70±7	29.1±6.4	Tread	COD (m); COT (sec)	BRJ	0.11 (mmol/kg)	1	60	-	-
Pekas et al. 2023	PAD	10 (40%)	70±9	29.3±6.6	Tread	6MW (m)	BRJ	0.11 (mmol/kg)	1	60	-	-
Schwarz et al. 2017	Angina	70 (74%)	67±8	28.6±4.0	Tread	TTE (sec)	$NaNO_3^-$	7	8.5	150	297.6±164.3	552±320
van der Avoort et al. 2021	PAD	18 (61%)	73±8	25.0±3.0	Tread	TTE (sec); COT (sec)	BRJ	6.5	1	180	494±110	1353±1316
Woessner et al. 2020	HFrEF	16 (94%)	63±14	31.8±8.4	Tread	VO_2Peak (ml/min/kg); TTE (sec)	BRJ	16	7	150	1084.3±471.8	342.1±234.3

Zamani et al. 2015	HFpEF	17 (88%)	66±9	36.4±5.4	Cycle	VO_2Peak (ml/min/kg); TTE (sec)	BRJ	12.9	1	180	-	-

Abbreviations: Pops. = the populations investigated in each study: HFrEF, heart failure with reduced ejection fraction, HFpEF, heart failure with preserved ejection fraction; PAD, peripheral arterial disease; and NIDC, non-ischemic dilated cardiomyopathy;. Modes = forms of exercise modality used to evaluate fitness: cycle, cycle ergometer; tread, treadmill walking ramp or fixed workload; and walk/walking, a walking field test. Variables = exercise outcomes assessed: TTE Submax, time to exhaustion during a fixed workload; VO_2 Submax, oxygen consumed during a fixed workload test; VO_2Peak, maximal oxygen consumed during an incremental test; TTE, time to exhaustion during an incremental maximal exercise test; 6MW, distance walked during a 6-minute walk, ISWT, distance walked during an incremental shuttle walk test; COD or COT, distance or time walked until claudication pain. Mode = NO_3^- supplementation used: $NaNO_3^-$, sodium nitrate, or BRJ, beet root juice. Days taken = the length of treatment in days. Last Dose = the time in minutes the last dose was consumed before post-testing.

Table S3. Pooled analysis excluding de Avoort et al. 2021.

TTE NO_3^- vs Placebo (excluding van de Avoort et al. 2021)

Model results

Estimate	SE	z-value	P value	Lower 95% CI	Upper 95% CI
0.0932	0.1237	0.7328	0.4637	-0.1518	0.3331

Test for heterogeneity

Q(df = 5) = 0.5558, p<0.9899

Study ID	D1	D2	D3	D4	D5	Overall
Kerley et al. 2016	+	+	+	+	+	+
Shepherd et al. 2015	+	+	+	!	+	+
Coggan et al. 2018	+	+	+	+	+	+
Eggebeen et al. 2016	+	+	+	+	+	+
Zamani et al. 2015	+	+	+	+	+	+
Friis et al. 2017	+	+	+	+	+	+
Kerley et al. 2018	+	+	+	+	+	+
Kenjale et al. 2011	+	+	+	+	+	+
Woessner et al. 2020	+	+	+	+	+	+
Curtis et al. 2015	+	+	+	+	+	+
Leong et al. 2015	+	+	+	+	+	+
Pekas et al. 2023	+	+	+	+	+	+
Coggan et al. 2015	+	+	+	+	+	+
Pavitt et al. 2022	+	+	+	+	+	+
Pekas et al. 2021	+	+	+	+	+	+
Ramick et al. 2021	+	+	+	+	+	+
van der Avoort et al. 2021	+	+	+	+	+	+
Hirai et al. 2017	+	+	+	+	+	+
Schwarz et al. 2017	+	+	+	+	+	+
Kerley et al. 2015	+	+	+	+	+	+
Shepherd et al. 2015	+	+	+	+	+	+
Beijers et al. 2018	+	+	+	+	+	+

+ Low risk

! Some concerns

- High risk

D1 Randomisation process

D2 Deviations from the intended interventions

D3 Missing outcome data

D4 Measurement of the outcome

D5 Selection of the reported result

Figure S1. Risk of Bias (ROB) 2 Bias Assessment

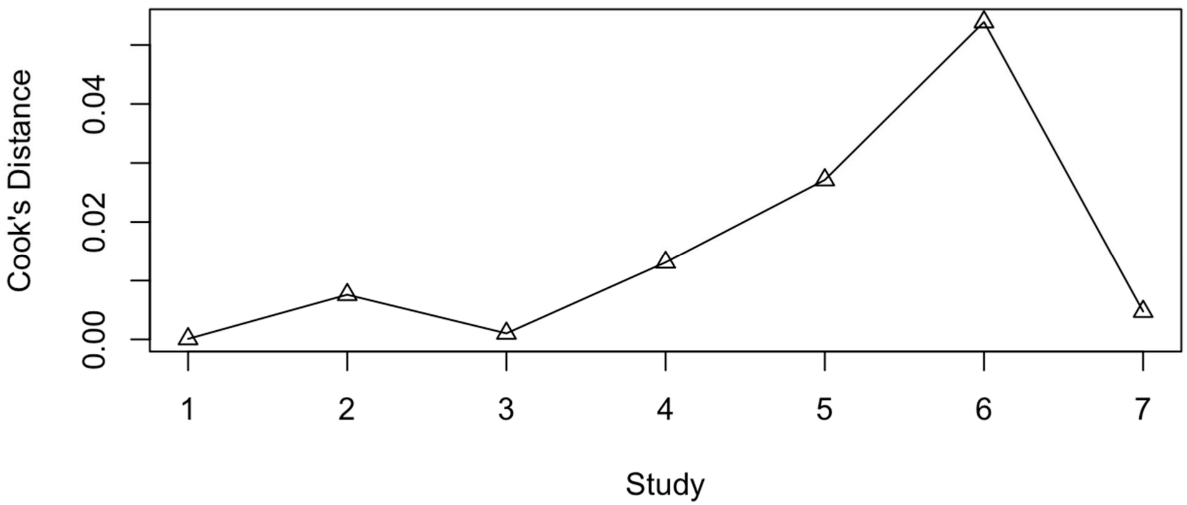

Figure S2. TTE Cook's Distance depicting Trials #5 (Woessner et al. 2020) and Trial #6 (Schwartz et al. 2017) as either outlying or influential trials.

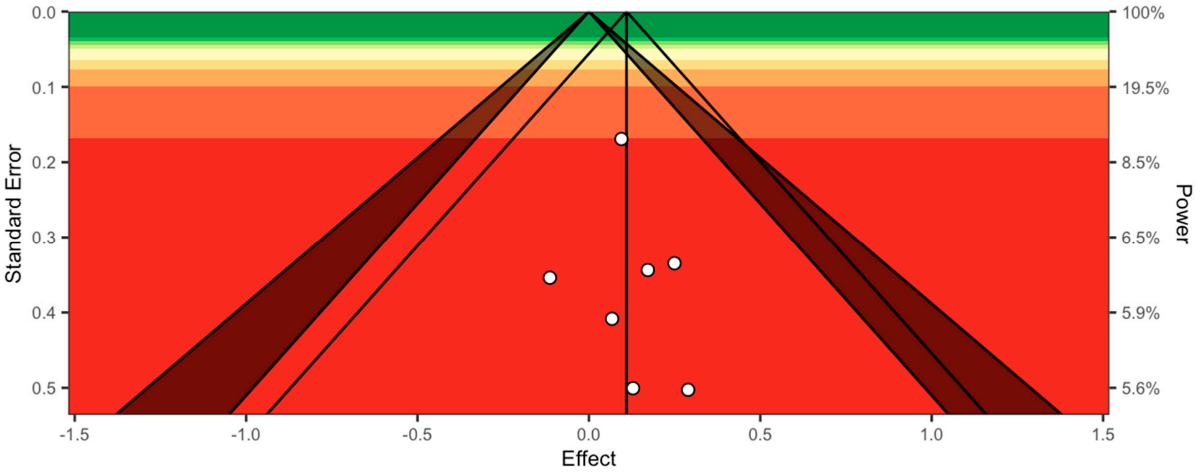

Figure S3. TTE Funnel Plot. The observed outcome is SMD which is plotted against the standard error.

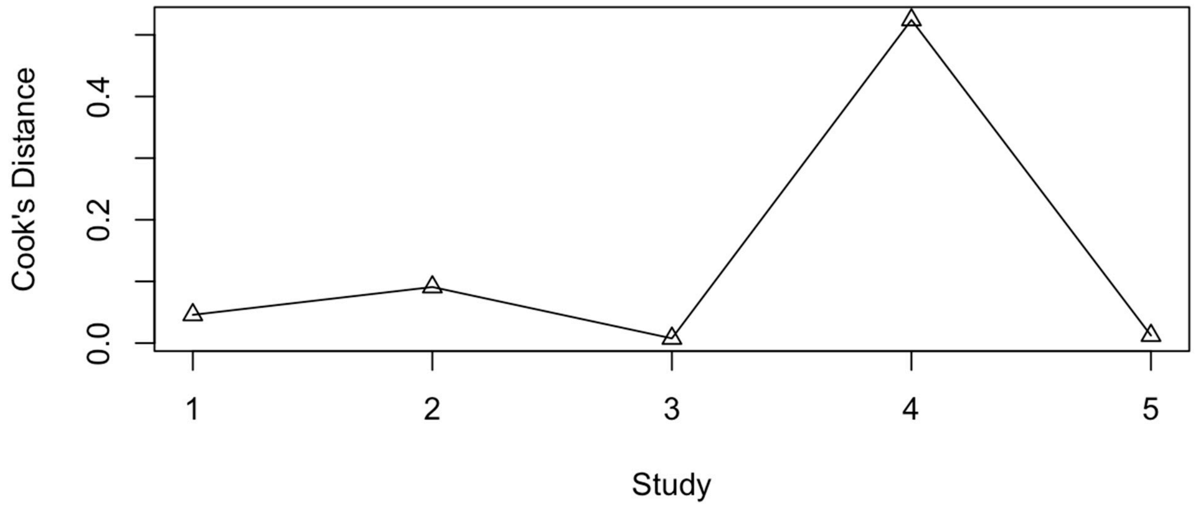

Figure S4. Submaximal TTE Cook’s Distance depicting Trial #4 (Eggenbeen et al. 2016 7d treatment trial) as an outlying or influential trial.

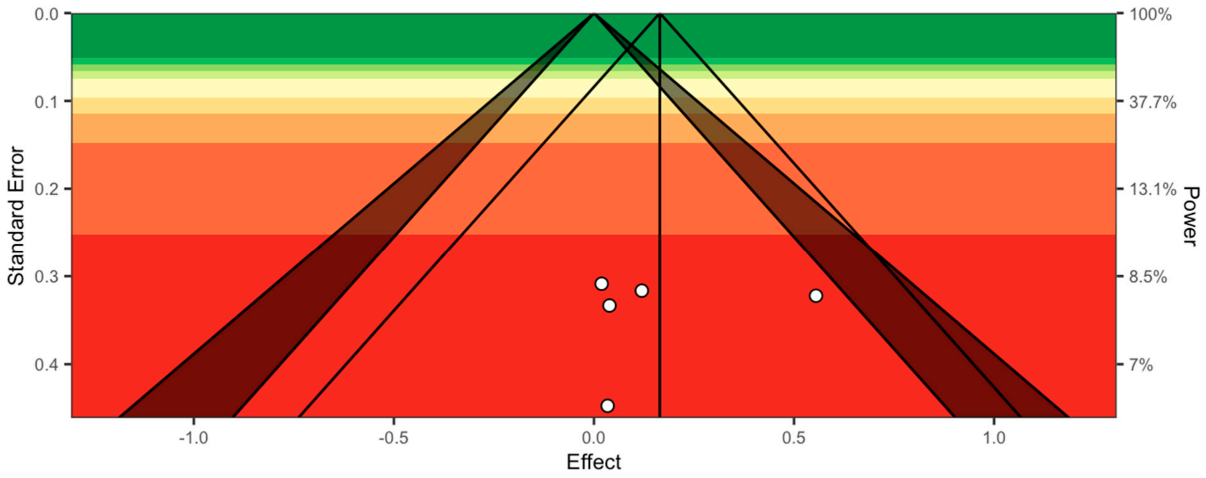

Figure S5. Submaximal TTE Funnel Plot. The observed outcome is SMD which is plotted against the standard error.

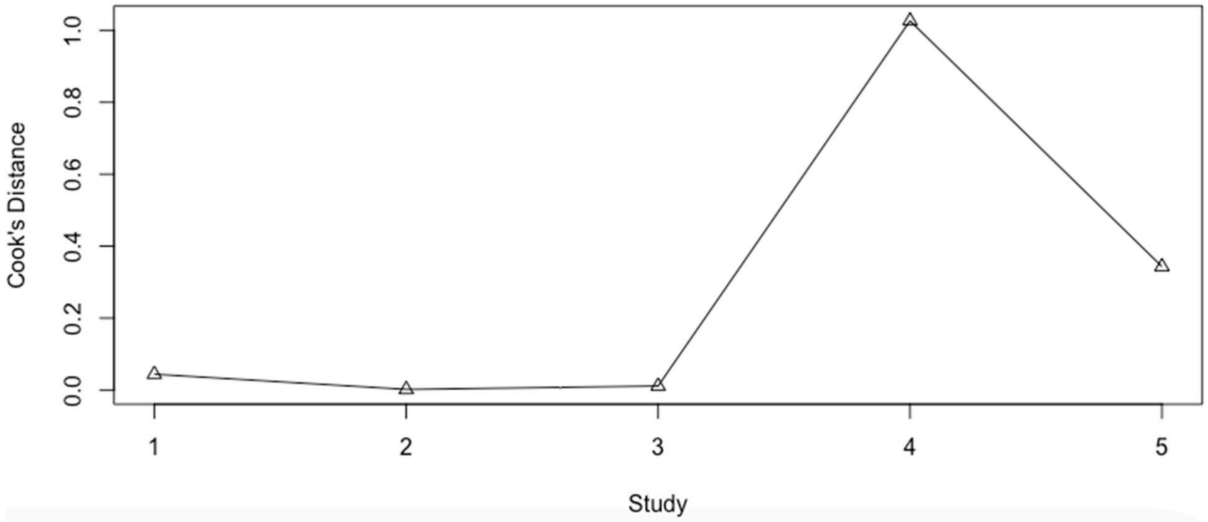

Figure S6. VO_2Peak Cook's Distance depicting Trial #4 (Woessner et al. 2020) as an outlying or influential trial.

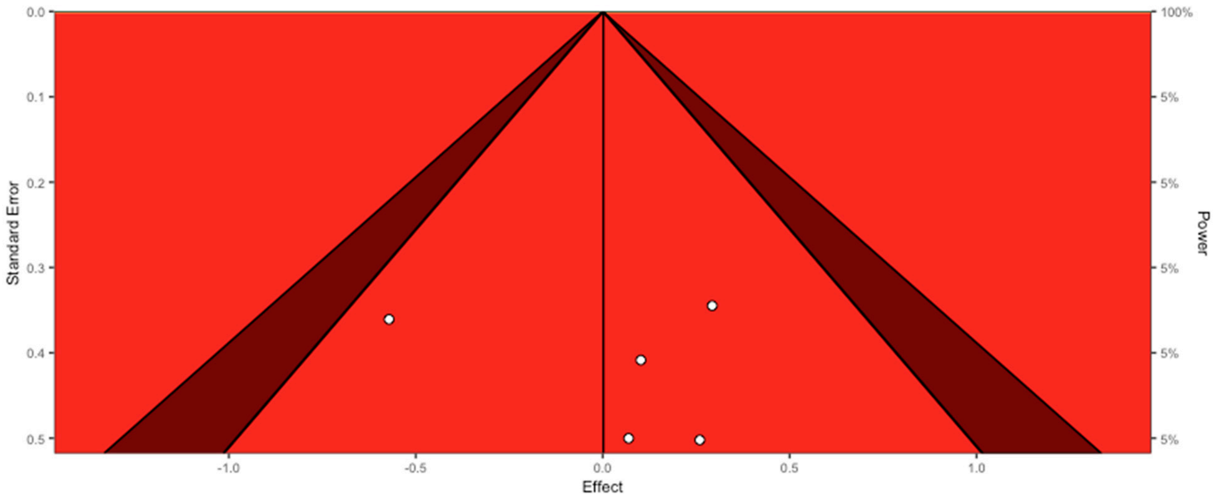

Figure S7. VO_2Peak Funnel Plot. The observed outcome is SMD which is plotted against the standard error.

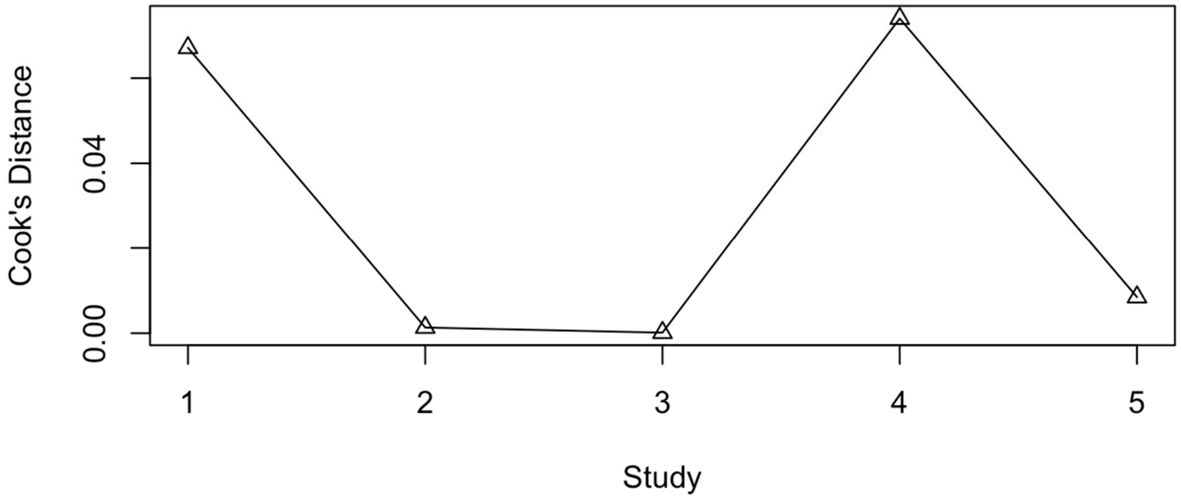

Figure S8. 6MW Distance Cook's Distance depicting Trials #1 (Coggan et al. 2015) and #4 (Shepherd et al. 2015) as outlying or influential studies.

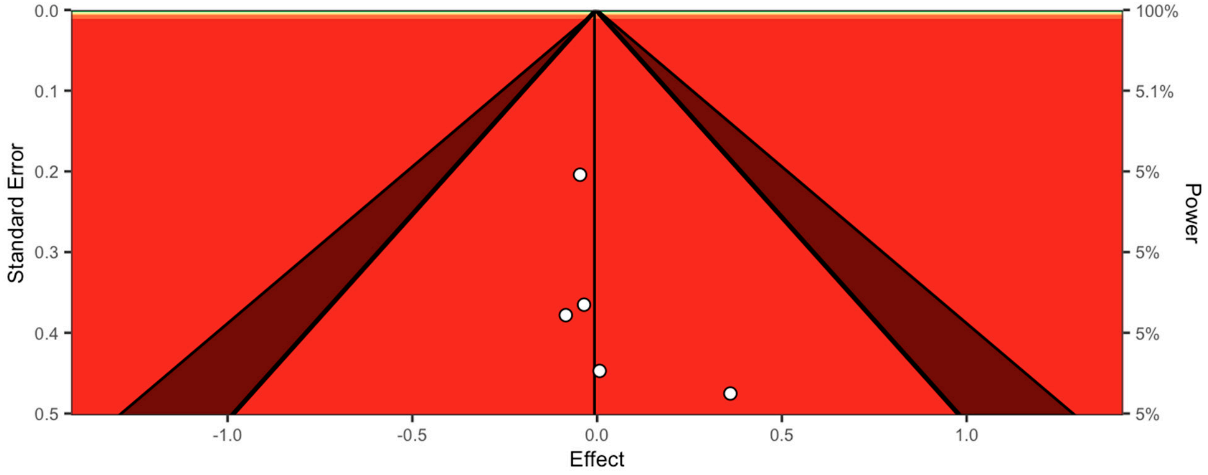

Figure S9. 6MW Distance Funnel Plot. The observed outcome is SMD which is plotted against the standard error.

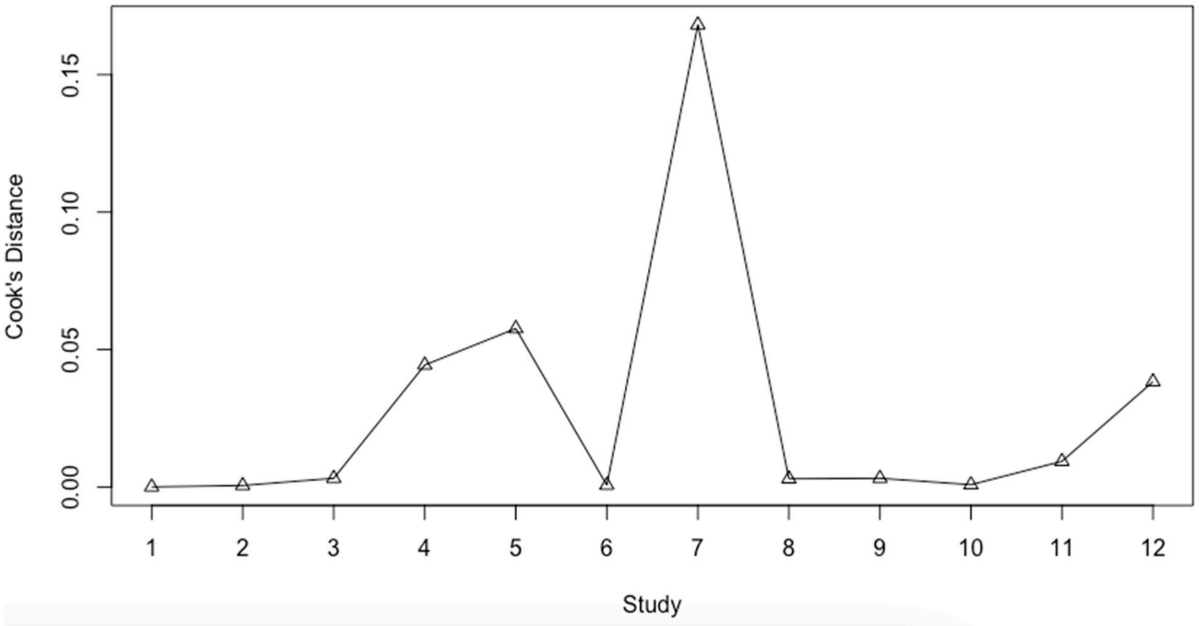

Figure S10. Timed Exercise Trials in CVD Cook's Distance depicting Trial #4 (Woessner et al. 2020), #5 (Schwarz et al. 2017), #7 (Eggebeen et al. 2016) and #12 (van der Avoort et al. 2021) as outlying or influential trials.

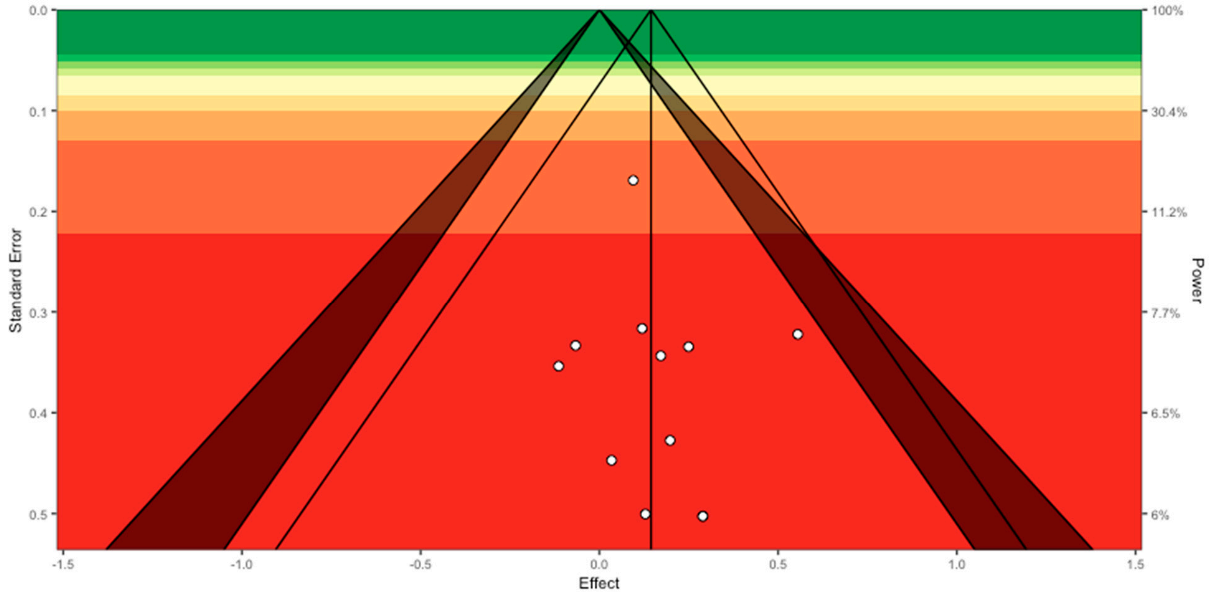

Figure S11. Timed Exercise Trials Funnel Plot. The observed outcome is SMD which is plotted against the standard error.

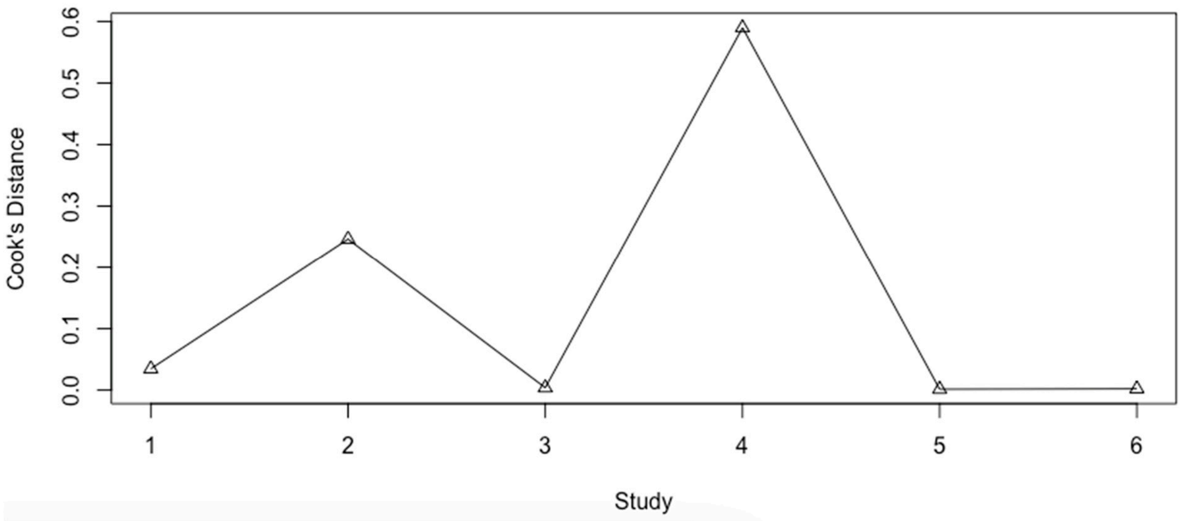

Figure S12. VO_2 in CVD Cook's Distance depicting Trial #4 (Woessner et al. 2020) as an influential trial.

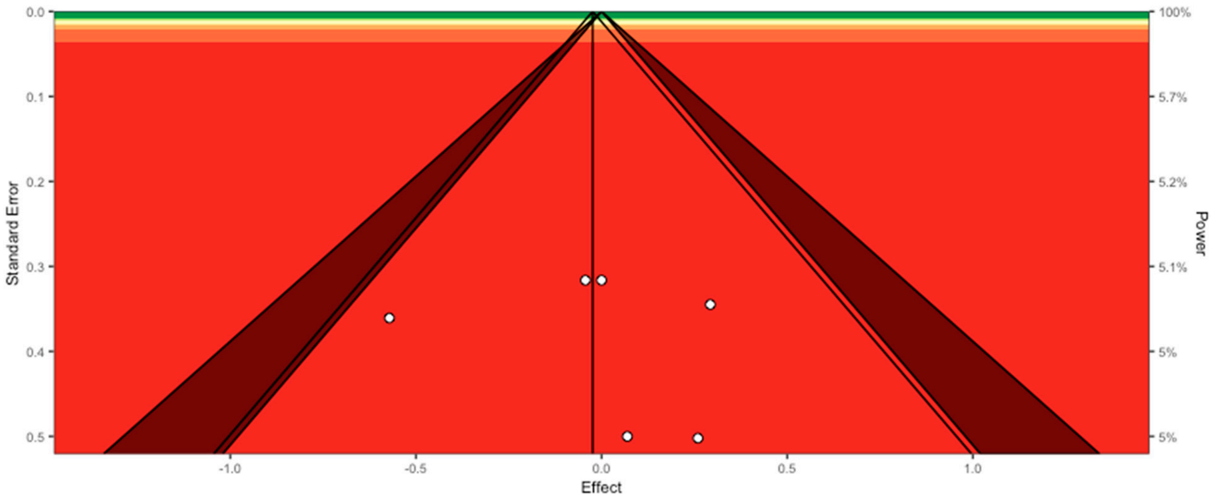

Figure S13. VO_2 Trials Funnel Plot. The observed outcome is SMD which is plotted against the standard error.

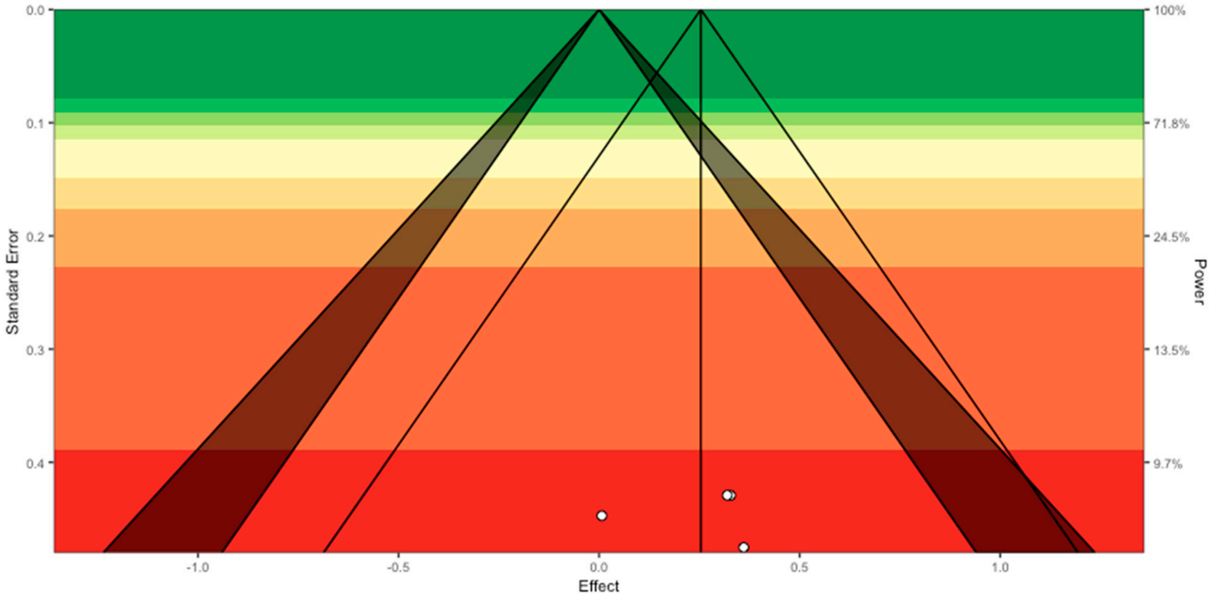

Figure S14. Distance Exercise Trials Funnel Plot. The observed outcome is SMD which is plotted against the standard error.
